# Development of a Clinical Global Impression of Change (CGI-C) and a Caregiver Global Impression of Change (CaGI-C) measure for ambulant individuals with Duchenne muscular dystrophy

**DOI:** 10.1186/s12955-021-01813-w

**Published:** 2021-07-26

**Authors:** Hannah Staunton, Claire Trennery, Rob Arbuckle, Maitea Guridi, Elena Zhuravleva, Pat Furlong, Ryan Fischer, Rebecca Hall

**Affiliations:** 1 Roche Products Limited, Welwyn Garden City, UK; 2 Adelphi Values, Patient-Centered Outcomes, Bollington, UK; 3 F. Hoffman-La Roche, Basel, Switzerland; 4 Parent Project Muscular Dystrophy, Hackensack, NJ USA

**Keywords:** Duchenne muscular dystrophy, Quality of life, Children, Adolescent, Clinical outcome assessment

## Abstract

**Background:**

In clinical trials for rare diseases, such as Duchenne muscular dystrophy, clinical outcome assessments (COA) used to assess treatment benefit are often generic and may not be sensitive enough to detect change in specific patient populations. Thus, there is a need for disease specific COAs that track meaningful change among individuals. When developing such measures, input from clinicians, caregivers and patients is critical for assessing clinically relevant concepts and ensuring validity of the measure.

**Method:**

The aim of this study was to develop two Duchenne-specific global impression items for use in clinical trials. The development of the Duchenne Clinical Global Impression of Change (CGI-C) and Caregiver Global Impression of Change (CaGI-C) was informed by findings from concept elicitation (CE) interviews with clinicians, caregivers and individuals with Duchenne. Through cognitive debriefing (CD) interviews, clinicians and caregivers evaluated draft CGI-C and CaGI-C items to ensure relevance and understanding of the items and instructions. Suggestions made during the CD interviews were incorporated into the finalized CGI-C and CaGI-C measures.

**Results:**

The symptoms most frequently reported by clinicians, caregivers and individuals with Duchenne were muscle weakness, fatigue, cardiac difficulties and pain. Regarding physical functioning, all three populations noted that small changes in functional ability were meaningful, particularly when independence was impacted. Caregivers and clinicians reported that changes in speed, endurance and quality of movement were important, as was improvement in the ability of individuals to keep up with their peers. A change in the ability to complete everyday activities was also significant to families. These results were used to create two global impression of change items and instruction documents for use by clinicians (CGI-C) and caregivers (CaGI-C). Overall, both items were well understood by participants. The descriptions and examples developed from the CE interviews were reported to be relevant and appropriate for illustrating different levels of meaningful change in patients with Duchenne. Modifications were made based on caregiver and clinician CD feedback .

**Conclusions:**

As part of a holistic measurement strategy, such COA can be incorporated into the clinical trial setting to assess global changes in relevant symptoms and functional impacts associated with Duchenne.

**Supplementary Information:**

The online version contains supplementary material available at 10.1186/s12955-021-01813-w.

## Background

Duchenne muscular dystrophy (Duchenne) is a rare genetic disorder that affects one in every 5500–6250 births worldwide [1]. Duchenne is an X-linked condition caused by deletions or mutations in the *DMD* (Duchenne muscular dystrophy) gene, which encodes the dystrophin protein [2, 3]. An absence of, or deficiency in, dystrophin protein results in progressive muscle degeneration [4].

In individuals with Duchenne, initial disease symptoms emerge in early childhood, with children aged 1–3 years experiencing delayed walking, difficulty with walking (e.g. atypical waddling gait or toe-walking), and/or frequent falls [1, 4, 5]. As children age, a steady decline in muscle function occurs, with many individuals losing ambulation and requiring a wheelchair by 8–14 years of age [1]. After the loss of ambulation, certain comorbid complications progress more rapidly, including scoliosis and muscular contractures [1]. By their late teenage years, most young people with Duchenne experience a decline in upper body and extremity functioning (e.g. moving and/or lifting the head and arms or gripping and picking up objects)—further reducing their independence and health-related quality of life (HRQoL) while increasing cardiovascular and respiratory complications [1, 6–8]. By 30–40 years of age, individuals typically die from cardiac or respiratory failure [9, 10].

Disease progression affects individuals’ physical, psychological, social and overall wellbeing, in addition to impacting the lives of families and caregivers [11]. A growing movement towards patient-centered care emphasizes the need for outcomes designed with the patient’s disease experience and perspective in mind [12, 13]. The US Food and Drug Administration (FDA) recognize the critical role patients, caregivers and clinicians play in developing specific clinical outcome assessments (COAs) for use as endpoints in clinical trials and, as a result, released a series of patient-focused drug development guidance documents [14, 16]. In a clinical trial setting, treatment benefit can be measured using a generic Clinical Global Impression of Change (CGI-C). In the CGI-C, clinicians are asked to rate the degree of change observed in a patient since the beginning of the study. Caregiver global impression of change items can also provide valuable insights regarding the level of treatment benefit experienced from an observer perspective. These items are typically rated on a seven-point scale from ‘very much improved’ to ‘very much worse’ [15]. However, the item is not disease specific and is used across indications. This leads to inconsistencies in the concepts considered when rating change and how change is rated among individual clinicians or caregivers leading to inter-rater variability.

For Duchenne, measures assessing clinically meaningful functional change are essential for evaluating the efficacy of an investigational treatment. Currently no disease-specific global impression of change items exists which directly assess the symptoms and functional abilities important to individuals with Duchenne from a patient-centered perspective. This study aimed to explore meaningful changes in symptoms and functional abilities of individuals with ambulatory Duchenne through qualitative interviews with the individuals, their caregivers and clinicians. This information was used to develop a Duchenne-specific CGI-C and Caregiver Global Impression of Change (CaGI-C) items, designed to assess disease-specific changes in global health status in the context of a clinical trial. These measures were included in the Phase 2/3 clinical trial (NCT03039686) of RG6206 (RO7239361) in boys with Duchenne.

## Methods

### Sample and recruitment

**Fig. 1:**
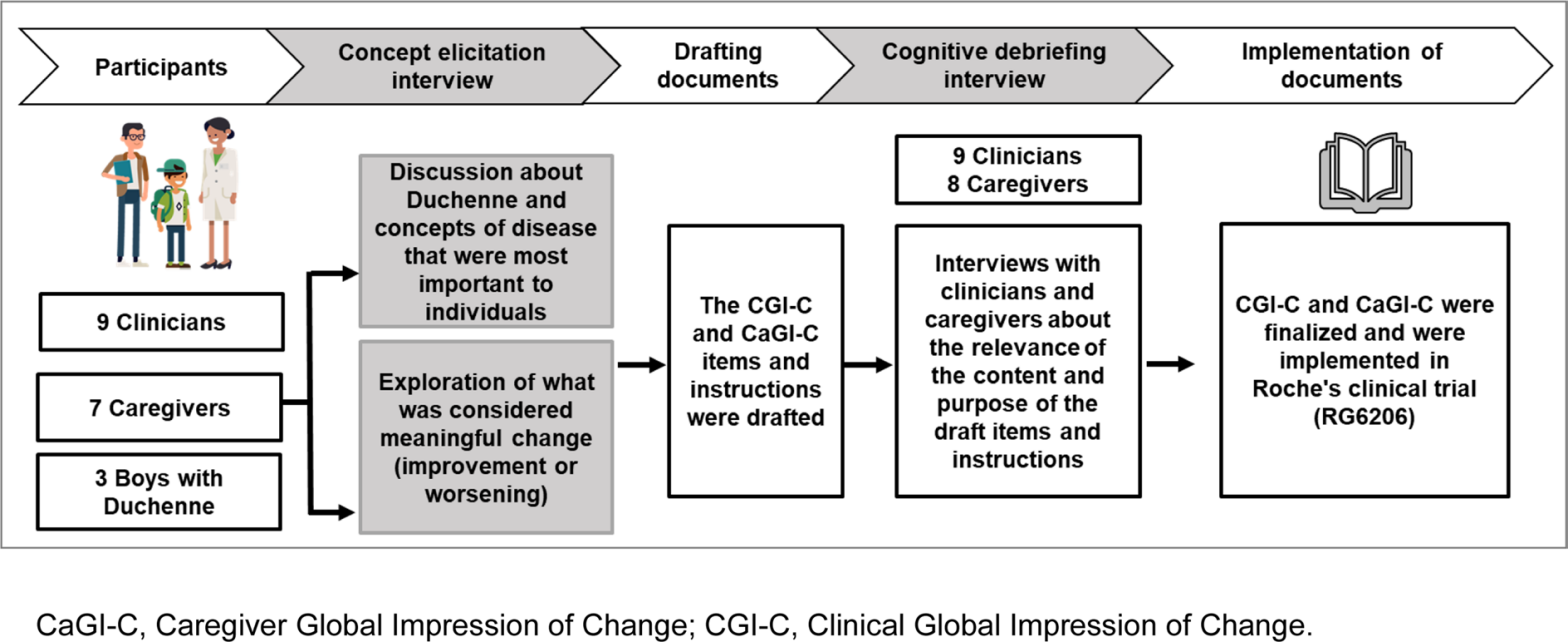
Overview of the development of CGI-C and CaGI-C items. CaGI-C, Caregiver Global Impression of Change; CGI-C, Clinical Global Impression of Change

In this non-interventional, cross-sectional, qualitative study, participants were recruited for concept elicitation (CE) interviews and cognitive debriefing (CD) interviews. An overview of the study and sample population is shown in Fig. 1. Nineteen participants, including clinicians, caregivers and Duchenne dyads (a pair that includes a caregiver and patient), were recruited for the CE interviews. CE is the process of collecting relevant concepts (e.g. symptom and impacts) that are important to the population of interest from relevant stakeholder perspectives (e.g. patients, caregivers, clinical experts) [16].

Seventeen participants, including caregivers and clinicians, were recruited for the CD interviews. Six of the clinicians and five of the caregivers who took part in the CE interviews also took part in the CD interviews. CD is the process of determining whether the content of a COA instrument (specifically the items, concepts under assessment, response options and recall period) are relevant to, and understood by respondents as intended by the developers [14]. Clinicians were identified using a third-party recruitment agency in the US. Two patient advocacy groups, Parent Project Muscular Dystrophy (US) and Action Duchenne (UK), assisted in identifying eligible caregivers and individuals with Duchenne to participate in the study.

Included in the study were ambulant boys (individuals able to walk unassisted for 10 m or more) aged 8–11 years at the time of the interview who were diagnosed with Duchenne and were receiving a stable dose of corticosteroids for 3 months prior to the interview date. Diagnosis of Duchenne was confirmed by caregiver-reported medical history (e.g. onset of clinical signs or symptoms before 5 years of age together with an elevated serum creatine kinase level observed before or after initial diagnosis) and by genotyping (only out-of-frame deletions allowed). Eligible caregivers included in the study were primary, unpaid caregivers of ambulant individuals with Duchenne aged 6–11 years. Clinicians included in the study were neurologists specializing in neuromuscular disorders who had a minimum of 5 years of experience in treating individuals with Duchenne.

### CE interviews

One-to-one CE interviews were conducted by trained qualitative researchers with clinicians (n = 9; 30 min), caregivers (n = 7; 45 min) and individuals with Duchenne (n = 3; 30 min). Face-to-face interviews were conducted with Duchenne dyads whenever possible. When face-to-face interviews were not possible, the interview was conducted via telephone. All clinician interviews were conducted via telephone.

A semi-structured interview guide was followed. The first half of these interviews comprised open-ended questions to facilitate spontaneous discussions. The concepts related to the symptoms and functional and HRQoL impacts associated with Duchenne were explored. Meaningful changes relating to these concepts (both improvement and worsening) in the context of a hypothetical investigational treatment were explored in the second half of the interview.

### Development of the draft CGI-C and CaGI-C

The findings from the CE interviews were used to develop an initial draft of the CGI-C and CaGI-C document, which contained the global impression of change item and associated instructions regarding their completion*.* Given that a generic global impression of change item exists, it was used as the framework for developing the Duchenne specific global impression item content [15]. To ensure specificity of the global impression of change item to Duchenne, the findings from the CE interviews formed part of a section titled ‘information to consider’, which was intended to advise clinicians and caregivers on the symptom and impact concepts to evaluate when assessing change. CE data was also used to develop the descriptions and supporting examples of the response options for each level of change (i.e. very much improved, much improved, minimally improved, no change, minimally worse, much worse and very much worse), as included in the document.

### CD interviews

The CD interviews were conducted by the same researchers who conducted the CE interviews and were performed individually with clinicians (n = 9; 30 min) and caregivers (n = 8; 30 min); no individuals with Duchenne participated in the CD interviews due to the focus on developing clinical and caregiver global impression items. A semi-structured interview guide was followed. A “think aloud” technique was utilized, where participants were asked to read each section of the draft document of the CGI-C or CaGI-C aloud, which contained the item and instructions for its completion, and provide verbal feedback on the document content. Clinicians and caregivers were asked detailed follow-up questions to evaluate their comprehension of the purpose of the CGI-C and CaGI-C document and its content, its relevance to individuals with Duchenne and its usability in the context of a clinical trial. Of note, due to significant revisions to the CaGI-C, the final interview with the eighth respondent involved debriefing of an updated measure, based on the prior seven interviews.

### Analysis of interviews

All interviews were audio-recorded, transcribed and entered into ATLAS [17] a software package designed to facilitate the storage, coding, and analysis of qualitative data.

CE interview transcripts were analyzed by trained qualitative researchers specializing in the development and validation of clinical outcome assessments. Thematic analysis is a qualitative research method that involves identifying, analyzing and reporting themes within data, using the patient’s language during the coding process [18]. Participant quotes pertaining to the main research objectives (i.e. symptoms, physical functioning, activities of daily living [ADL] and meaningful change) were highlighted and assigned corresponding concept codes.

CD interview transcripts were analyzed by the same trained qualitative researchers using a framework approach. For this, dichotomous codes were assigned to each item, instruction, response option and recall period of the CGI-C and CaGI-C discussed by participants to denote whether it was relevant/not relevant to the lived experience of Duchenne, understood/not understood by participants, and easy/difficult to complete. As outlined in the FDA PFDD guidance 3 document, an understanding of the COA content is critical [14]. Further codes to indicate why specific response options were chosen, how the content applied to the experience of Duchenne from the caregiver or clinician perspective, and suggestions for wording or formatting changes were also applied to the CD data.

### Finalizing CGI-C and CaGI-C

Findings from the CD interviews were used to inform updates to the CGI-C item and CaGI-C items and their instructions. During this process, feedback from clinicians and caregivers on the understanding, relevance and feasibility of the use of CGI-C and CaGI-C items and instructions, and suggested wording changes were considered with the aim of improving the clarity of content for readers, ease of implementation in the context of a clinical trial, and the selection of an appropriate response option denoting level of perceived change. The information considered from the CE/CD interviews that informed the final content of the CGI-C and CaGI-C is listed in Tables 5 and 6.

### Ethical approval

This study was approved by Copernicus Independent Review Board (ADE1-18-027). All participants provided their consent/assent prior to the conduct of any research-related activities.

## Results

### CE sample: Participant demographics

**Fig. 2:**
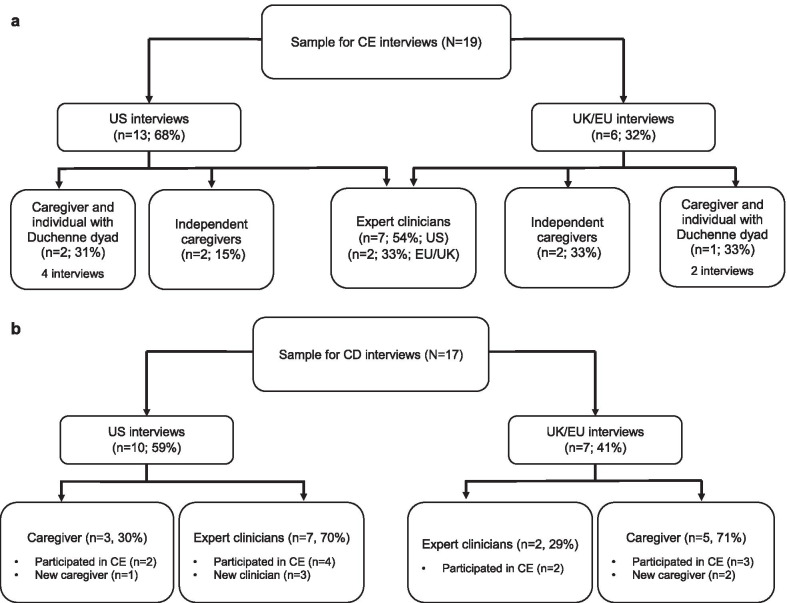
Overview of the concept elicitation (CE, **a**) and cognitive debriefing (CD, **b**) sample

The majority of the participants who took part in the CE interviews were based in the US (Fig. 2a). US-based participants included seven clinicians, two independent caregivers and two Duchenne dyads. The remaining six participants were from the UK/EU. Of these participants, two were clinicians, two were independent caregivers, and two were part of a Duchenne dyad.

Clinicians were mainly pediatric neurologists who had been in practice for over 15 years (Table 1). Most clinicians managed the care of ≥ 25 individuals with Duchenne. All caregivers were the parents of individuals aged 6–10 years with Duchenne (Table 2).

**Table 1:** Demographics of the clinician sample

Demographic	CE sample	CD sample
	Total no. of clinicians (n = 9)	Total no. of clinicians (n = 9)
Job title, n (%)		
Neurologist	3 (33.3%)	5 (55.6%)
Pediatric neurologist	6 (66.7%)	4 (44.4%)
Time in role, n (%)		
Range (years)	6–25	8–30
Less than 10 years	3 (33.3%)	2 (22.2%)
10–15 years	1 (11.1%)	1 (11.1%)
Over 15 years	5 (55.6%)	6 (66.7%)
Years treating individuals with Duchenne, n (%)		
Range (years)	8–30	8–30
Less than 10 years	1 (11.1%)	1 (11.1%)
10–15 years	3 (33.3%)	3 (33.3%)
Over 15 years	5 (55.6%)	5 (55.6%)
Individuals currently managing with Duchenne, n (%)		
0–25 individuals	3 (33.3%)	5 (55.6%)
26–50 individuals	5 (55.6%)	3 (33.3%)
51–75 individuals	0	0
76–100 individuals	1 (11.1%)	1 (11.1%)

CD, cognitive debriefing; CE, concept elicitation

**Table 2:** Demographics of the caregiver sample

Demographic	CE sample	CD sample
	Total no. of caregivers (n = 7)	Total no. of caregivers (n = 8)
Gender, n (%)		
Male	1 (14.3%)	1 (12.5%)
Female	6 (85.7%)	7 (87.5%)
Age of individual with Duchenne cared for n, (%)		
6–7 years	2 (28.6%)	3 (37.5%)
8–9 years	2 (28.6%)	3 (37.5%)
10 years	3 (42.8%)	2 (25.0%)
Caregiver race, n (%)		
White	6 (85.7%)	6 (75.0%)
Asian	0	1 (12.5%)
Hindu*	1 (14.3%)	1 (12.5%)
Relationship with individual cared for, n (%)		
Parent/guardian	7 (100.0%)	8 (100.0%)
Work status, n (%)		
Working full time	6 (85.7%)	5 (62.5%)
Working part time	1 (14.3%)	1 (12.5%)
Full-time homemaker	0	1 (12.5%)
Occasional consultancy work	0	1 (12.5%)
Level of education		
High school diploma	1 (14.3%)	1 (12.5%)
Some years of college	2 (28.6%)	3 (37.5%)
Cert program	1 (14.3%)	0 (0%)
College or university	2 (28.6%)	1 (12.5%)
Graduate or professional degree	1 (14.3%)	3 (37.5%)

*Self-reported race as Hindu

CD, cognitive debriefing; CE, concept elicitation

Clinical and demographic characteristics were collected for all seven individuals with Duchenne who either participated in the study as part of a Duchenne dyad (n = 3) or were represented by a caregiver (n = 4) (i.e. independent caregiver interview). The majority of individuals with Duchenne who participated in the study were represented by a caregiver and were diagnosed before the age of 6 and experienced first symptoms associated with Duchenne between 0 and 5 years (Table 3). All individuals with Duchenne were ambulatory boys based on the study’s definition.

**Table 3:** Demographics of the individuals with Duchenne

Demographic	CE sample
	Total no. of individuals interviewed (n = 7*)
Age of individual with Duchenne (%)	
6–7 years	2 (28.6%)
8–9 years	2 (28.6%)
10 years	3 (42.8%)
Age diagnosed with Duchenne, n (%)	
Under 5 years old	4 (57.1%)
Between 6 and 10 years old	3 (42.9%)
Age experienced symptoms of Duchenne, n (%)	
0–5 years old	5 (71.4%)
6–10 years old	2 (28.6%)
Can the individual with Duchenne walk? n (%)	
Yes	7 (100.0%)
No	0

*Three individuals with Duchenne participated in the CE interviews and four caregivers were interviewed on behalf of the individual with Duchenne

CE, concept elicitation

### CD sample: Participant demographics

Seventeen participants were recruited for the CD interviews, with over half of participants from the US (Fig. 2b). Six clinicians who participated in the CE interviews also participated in the CD interviews. Three new clinicians were recruited, resulting in a total of nine clinicians. Five caregivers who participated in the CE interviews also participated in the CD interviews. Three new caregivers were recruited, resulting in a total of eight caregivers.

Nearly an equal distribution of pediatric neurologists and generalist neurologists were represented (Table 1). Over half of the clinicians had been in practice for over 15 years and managed the care of up to 25 individuals with Duchenne. Among the caregiver sample, all were the parents of individuals aged 6–10 years with Duchenne, as shown in Table 2. The highest level of education achieved by caregivers ranged from a high school diploma to a graduate or professional degree.

### CE interviews

During open-ended discussions, clinicians described 10 symptoms experienced by individuals with Duchenne. Muscle weakness (n = 9/9) and fatigue (n = 6/9) were reported as the key defining features of the disease. Muscle weakness affected the physical functioning of the proximal muscles in both the lower and upper extremities. The next most frequently reported symptom was cardiac difficulties (n = 4/9). Caregivers and individuals spontaneously elicited five symptoms when asked about symptoms of Duchenne. Similar to clinician reports, muscle weakness and fatigue/tiredness were the most frequently reported symptoms. This was followed by pain/discomfort, muscle tightness/stiffness and constipation in order of frequency.

The impacts of Duchenne on physical functioning were discussed during the CE interviews from the perspective of clinicians, caregivers, and individuals with Duchenne. A total of 17 impacts on physical function were discussed primarily in context of the concepts assessed by the North Star Ambulatory Assessment [19]. Walking, climbing stairs, standing up from sitting on a chair, and standing up from the floor were among the most reported impacts on physical functioning. These impacts were chosen as supporting examples and were included in the final item (CaGI-C) and/or instruction document (CGI-C). The most frequently raised meaningful improvements and worsening in relation to these concepts are described in Additional file 1.

**Table 4:** Supporting concept elicitation quotes from caregivers relating to meaningful improvements and meaningful worsening in activities of daily living (ADL)

Concept	Supporting quote on meaningful improvement	Supporting quote on meaningful worsening
**Washing**	“It would be an improvement for him to be able to **do it all by himself, the whole, the whole get in, wash up, and get out** and get dressed would be a big improvement.” (caregiver)	“Not being able **to stand in the shower** that would be way **worse**.” (caregiver)
**Dressing**	“Well he can do it, just—doing it with less struggle.” (caregiver)	“So a worsening would be that he can’t stand up to do it **comfortably**.” (caregiver)
	“Figuring out how to keep him **independent** and able to get changed himself.” (caregiver)	“**When he’s not able to lift his arms at all** to kind of help me get his shirt on.” (caregiver)
**Eating and drinking**	“Probably being **more effective at cutting** up his own food.” (caregiver)	“I suppose that sort of gradual change, you know, **when he can't actually cut any food at all**.” (caregiver)

With regard to the ability to walk, individuals with Duchenne, caregivers and clinicians described that walking longer distances without becoming fatigued would be considered a meaningful improvement. Requiring more assistance from supportive devices such as walking frames and devices, walking a shorter distance and walking on toes were all considered impacts constituting meaningful worsening by clinicians and caregivers. Caregivers also described feeling different to peers as being evidence of meaningful worsening.

All participants described some level of difficulty with ascending and descending stairs and all groups described that climbing stairs more quickly constituted meaningful improvement. Clinicians perceived the increased need for assistance while climbing stairs as meaningful worsening. Caregivers described meaningful worsening as losing the ability to climb stairs and the use of alternative techniques such as crawling to ascend the stairs.

Clinicians, caregivers and individuals with Duchenne all considered using less effort to stand from sitting or the ability to stand from lying down as meaningful improvements. Clinicians defined less effort as requiring little to no arm support to sit up (standing from sitting on a chair) or requiring less involvement of all four limbs (e.g. via Gowers’ movement) (standing up from the floor ). It was agreed upon by all groups that losing the ability to stand either from sitting or lying down was clear evidence of meaningful worsening, while needing more assistance was also considered to be an important indicator of meaningful worsening.

The impacts of Duchenne on ADL and what represents a meaningful change were discussed from the perspective of clinicians, caregivers and individuals with Duchenne (Table 4). Clinicians described washing (showering/bathing) and dressing as difficult for individuals with Duchenne due to muscle weakness in the arms and legs. All three populations considered washing independently without assistance from others or the use of adaptive environmental aids (e.g. handrails or hoists in the home) a meaningful improvement. In addition, all three groups also considered dressing independently or with reduced or no assistance from others a meaningful improvement. Caregivers and individuals reported any loss of independence or the requirement of assistance pertaining to bathing and showering, dressing, or lifting food to mouth and being able to use cutlery and cut up food as indicators of meaningful worsening.

While the focus of the interviews was on symptoms and functional abilities, caregivers and individuals also spontaneously described other concepts such as emotional and social impacts, including themes related to sadness, feeling different from peers, social isolation and a lack or loss of independence.

**Fig. 3:**
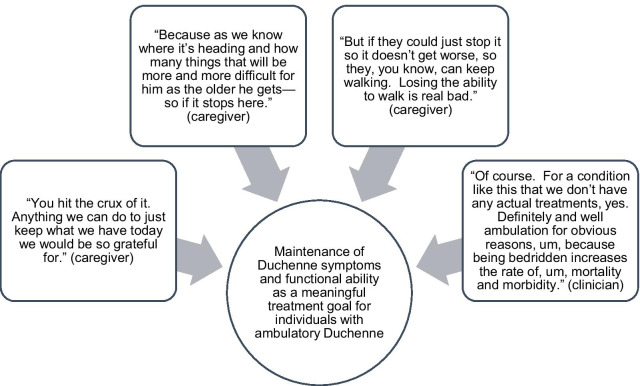
Maintenance of current functioning would be a meaningful treatment goal, from the perspective of caregivers and clinicians as reported in the concept elicitation interviews

Due to the progressive nature of the disease, both caregivers (n = 6/6 asked) and clinicians (n = 9/9) emphasized that maintenance of existing functional ability was a meaningful treatment goal for individuals with Duchenne (Fig. 3).

### Drafts of the CGI-C and CaGI-C

Preliminary CGI-C and CaGI-C items and instruction documents were drafted based on the findings from the CE interviews. The CGI-C item, which consists of a global question for clinicians and seven response options (i.e. very much improved to very much worse), was created based on the existing global impression of change item used by Guy et al. [15]. The CE data was used to tailor the remainder of the content to be specific to Duchenne (see Tables 5 and 6 for an overview of key findings). The first draft of the CGI-C contained a title, an explanation of the purpose of the document (e.g. to ensure standardization across raters with regard to the concepts to consider when evaluating change), information on how to rate change, including concepts to consider (e.g. those defined as important based on the CE interviews), and a global impression of change item with response options based on the original seven-point scale (1. very much improved, 2. much improved, 3. minimally improved, 4. no change, 5. minimally worse, 6. much worse, 7. very much worse). The information on how to rate change, including concepts to consider, was focused on assessing the patient’s clinical status (symptoms and functional ability) and consideration of whether a meaningful impact on daily life or wellbeing had occurred. The focus on symptoms and functional abilities were considered to be appropriate for clinicians given the observable and proximal nature of these concepts in addition to the fact that these concepts were frequently raised in the CE interviews and were therefore core concepts to Duchenne. Findings from the CE interviews were also used to develop vignettes of hypothetical individuals with Duchenne and information on how to rate an individual based on the seven-point scale. The activities that were deemed difficult in relation to motor ability and the associated consequences in daily life (i.e. improving or worsening on the activity in question) informed the creation of these vignettes. These vignettes were incorporated into the CGI-C draft instruction document.

**Table 5:** Summary of key sections across the CGI-C training document and final item/instruction document and associated CE/CD data

Section of CGI-C	Feature of detailed training document	Feature of top line item/instruction document	CE content that informed section	Key CE findings	CD content that informed section	Key CD findings
Title and purpose of CGI-C instruction document	✓	✓	N/A: title and purpose based on existing CGI-C principles [15]		Assessed understanding of the title, instructions and the relevance of a Duchenne-specific CGI-C	All clinicians (9/9) understood the title and 8/9 understood the purpose of the CGI-C All clinicians (9/9) considered the content relevant All clinicians (9/9) reported that the inclusion of information in the training document relating to how the CGI-C instructions had been developed (i.e. involving individuals, caregivers and neurologists) was informative and supported its credibility
Duchenne CGI-C definition and CGI-C items and response options	✓	✓	N/A: definition based on existing CGI-C principles [15]		N/A: definition based on existing CGI-C principles [15]	All clinicians (9/9) understood the Duchenne CGI-C definition and 7/8 considered the focus on symptoms and functional ability as relevant to the assessment of ‘overall health’ Several clinicians (4/9) advocated the use of the phrase ‘clinical status’ to represent symptoms and functional ability over the phrase ‘overall health’ as the latter was considered too broad The majority of clinicians asked (5/7) understood the Duchenne CGI-C item wording and 4/5 asked thought it was easy to complete
Information to consider when completing the CGI-C	✓	✓	Symptoms, physical functioning and ability to perform ADL were the key themes that arose as core to Duchenne in the CE clinician data	10 symptoms raised (muscle weakness, fatigue cardiac symptoms, enlarged calves, respiratory problems, muscle stiffness/tightness, contractures, pain, scoliosis and gastro-intestinal issues) 15 physical functioning activities raised (standing still, standing on heels, standing on one leg, standing up from a chair, standing up from lying on the floor, standing up from sitting on the floor, lifting head, walking, stepping onto box, stepping off of box, hopping, jumping, running, climbing stairs, difficulties with upper limb functioning) Five ADL difficulties were raised (bathing/showering, toileting, dressing, brushing hair, brushing teeth)	Assessed understanding of the instructions	The majority of clinicians (7/8) understood the ‘information to consider’ section Several of the clinicians asked (4/8) reported that they would improve the clarity of the ‘symptom’ domain in the ‘information to consider’ section by including key symptoms in addition to muscle weakness. The symptoms pain and fatigue were added to this section.
CGI-C determining change in response category descriptions	✓	✓	Descriptions of change category information was informed by the CE data where there was a focus on any change being meaningful	Meaningful improvement and worsening focused on symptoms and functional ability;a key theme was that any loss or gain of independence that led to changes in the need or level of assistance was important. In addition, the following themes emerged: Change in level of effort and confidence when carrying our activities Changes in speed, endurance and quality of movements	Assessed understanding of the instructions and item including descriptions	The majority of clinicians asked understood the descriptions of each level of change: ‘very much worse’ (9/9), ‘much worse’ (8/9), ‘minimally worse’ (9/9), ‘no change’ (8/8), ‘minimally improved’ (8/8), ‘much improved’ (7/7), ‘very much improved’ (6/6)
CGI-C determining change response category example	✓	✗	The examples were based on the themes raised by clinicians in the CE interviews and focused on symptoms such as muscle weakness, pain and physical functioning, as well as the amount of assistance or time required to complete a task		Assessed understanding of the instructions, domains and the relevance of the level of change descriptions and examples	The majority of clinicians asked considered the descriptions of each level of change to be relevant: ‘very much worse’ (8/9), ‘much worse’ (9/9), ‘minimally worse’ (5/5), ‘no change’ (7/8), ‘minimally improved’ (5/5), ‘much improved’ (5/5), ‘very much improved (4/5) All of the clinicians asked understood the examples of each level of change: ‘very much worse’ (9/9), ‘much worse’ (9/9), ‘minimally worse’ (9/9), ‘no change’ (6/6), ‘minimally improved’ (8/8), ‘much improved’ (6/6), ‘very much improved’ (5/5) The majority of clinicians asked consider the examples for each level of change to be relevant: ‘very much worse’ (8/9), ‘much worse’ (9/9), ‘minimally worse’ (8/9), ‘no change’ (7/8), ‘minimally improved’ (5/5), ‘much improved’ (8/8), ‘very much improved’ (4/5)
Example of CGI-C score vignettes	✓	✗	The vignettes were informed by the CE data. Changes in standing up from sitting and walking and the associated consequences in daily life of improving or worsening on these functions, informed the creation of the vignettes	Standing up from sitting on the floor and walking were of key importance clinically and thus chosen for the vignettes (see Additional file 1)	Assessed understanding of the vignettes	The majority of clinicians asked (6/7) thought the vignettes were useful, agreed with the assigned level of change rating (3/5) and considered the treatment notes relevant to Duchenne (2/2)

CE, concept elicitation; CD, cognitive debriefing; CGI-C, Clinical Global Impression of Change

**Table 6:** Summary of key sections across the CaGI-C item/instruction document and associated CE/CD data

Section of CaGI-C	Feature of item/instruction document	CE content that informed section	Key CE findings	CD content that informed section		Key CD findings
Title and purpose of CaGI-C instruction document	✓	N/A: Title and purpose based on existing CGI-C principles [15]			Assessed understanding of the instructions and the relevance of a Duchenne-specific CaGI-C	The majority of caregivers (5/7) understood the title and purpose of the CaGI-C All caregivers (7/7) reported that the inclusion of information relating to how the CaGI-C had been developed was relevant and informative One caregiver reported that the title and ‘purpose of the instructions’ section could be simplified to improve clarity, specifically relating to the time period over which changes should be considered (e.g. since the start of the clinical trial)
CaGI-C change response category descriptions (i.e. very much improved to very much worse)	✓	Selected domains chosen based on CE themes (symptoms, physical ability, ability to perform daily activities, social life, emotions and mental wellbeing and overall health) Descriptions of change category was informed by CE data indicating the meaning of improvement/worsening	Five symptoms raised (muscle weakness, fatigue, pain, muscle stiffness/tightness, constipation) 17 physical functioning activities raised (standing still, standing on heels, standing on one leg, standing up from a chair, standing up from lying on the floor, standing up from sitting on the floor, lifting head, walking, stepping onto box, stepping off of box, hopping, jumping, running, climbing stairs, bending over, exercise, difficulties with upper limb functioning) 10 ADL difficulties were raised (washing, dressing, eating/drinking, toileting, getting in/out of car, getting in/out of bed, brushing hair, brushing teeth, stretching and drawing/writing) 10 emotional concepts (feeling different to peers, reduced independence/autonomy, sadness/depression, worry/anxiety, feeling self-conscious/embarrassed, anger/frustration, reduced confidence, stress, maladaptive thoughts, irritability) Six social concepts (difficulties participating in social activities, difficulties during breaks/recess, need for modified activities/lessons, missed activities/school due to illness/medical appointments, limited mobility and obstacles outside of the home, marginalization/bullying) Three cognitive concepts raised but not included as a domain due to lack of relevance One behavior and one sleep concept raised but not included as domains due to lack of relevance A key theme was that any loss or gain of independence that led to changes in assistance was important. In addition, the following themes emerged: Change in level of effort and confidence Changes in speed, endurance and quality of movements Improvements in abilities associated with keeping up with peers	Assessed understanding of the change categories and response categories and the relevance of the descriptions		All the caregivers (7/7/) understood the description of each level of change (‘very much worse’, ‘much worse’, ‘minimally worse’, ‘no change’, ‘minimally improved’ and ‘very much improved) The majority of caregivers considered the descriptions of each level of change to be relevant: ‘very much worse’ (7/7), ‘much worse’ (6/7), ‘minimally worse (7/7), ‘no change’ (6/7), ‘minimally improved’ (6/7), ‘much improved’ (7/7/) and ‘very much improved’ (7/7)
Duchenne CaGI-C items and response options	✓	Selected domains selected based on CE themes (symptoms, physical ability, ability to perform daily activities, social life, emotions and mental wellbeing and overall health)		Assessed understanding of the instructions and items, domains and the relevance of the examples		All caregivers (7/7) understood the original global question (i.e. taking into account all of the individuals’ DMD symptoms and overall quality of life, how would you rate the change in his overall health since the start of this clinical trial? Please select one response only) Two caregivers indicated this question would be difficult to answer, leading to thinking about Duchenne in its entirety. This led to the creation of domain-level items, thus narrowing the concepts caregivers had to think about in relation to each item Updated domain CaGI-C: the final caregiver understood all items and assisted with the modification of the examples to ensure relevance (e.g. including strength as an example in symptoms)

CaGI-C, Caregiver Global Impression of Change; CE, concept elicitation; CD, cognitive debriefing

For the draft CaGI-C item, similar to the CGI-C, CE data was used to inform the content (see Table 6). The caregiver was required to consider the amount of change in overall health based on the symptoms, physical ability, ability to perform daily activities, social life, emotions, and mental wellbeing, of the individual with Duchenne when making their assessment of change. The rationale for focusing on both proximal (i.e. symptoms, physical ability, performing ADL) and distal concepts (social, emotional and mental wellbeing) stemmed from the importance of assessing the holistic disease experience from a caregiver perspective and also due to the observable nature of these concepts to caregivers who provide constant support [20, 21]. Moreover, in the interviews, caregivers described both proximal (e.g. physical abilities and ability to perform ADL) and distal impacts (such as social and emotional challenges); as such, these domains were considered important to include. A description of each change category (i.e. very much improved to very much worse) was developed based on the symptoms and impacts elicited from the interviews and the discussions around meaningful changes for these concepts.

### CGI-C document cognitive debriefing

The draft CGI-C item and instructions document were understood by all clinicians (see Table 5). The descriptions and examples for measuring the different levels of change in Duchenne were reported as relevant and appropriate. Minor revisions were made based on feedback provided by clinicians and included word changes that improved clarity or the selection of more appropriate examples of level of change (e.g. changing an example focused on the ability to ‘stand on one leg’ to one focused on ‘walking’ ability, which was more applicable to an individual’s daily life).

Due to feedback from clinicians, the instructions for the CGI-C item were split across two separate documents to shorten the length and to streamline information. The first document consisted of a single top-line item: “*Taking into account all aspects of the individual’s Duchenne symptoms and functional ability, how would you rate the change in clinical status for this individual since the start of the study? Please select one response only,*” with instructions on information to consider when rating change (see Additional file 2). In the second document, a more detailed scoring guideline with examples and vignettes of meaningful change was created, which was intended for use as a training document.

### CaGI-C document cognitive debriefing

The CaGI-C item and instructions were understood by caregivers (Table 6). Many caregivers reported the examples of the symptoms and physical functioning impacts used to illustrate the different levels of change in individuals with Duchenne were relevant and consistently understood. Minor revisions were made to improve wording clarity or to select more appropriate examples.

Caregivers and individuals with Duchenne emphasized the heterogeneity of the symptoms and impacts experienced by individuals with Duchenne and there was a suggestion that the domains which constitute overall health (symptoms, physical ability, ability to perform ADL, social life, and emotions and mental wellbeing) should be rated separately. In line with this, one caregiver specifically stated that the single global item—“*Taking into account all of the individual’s Duchenne symptoms and overall quality of life, how would you rate the change in his overall health since the start of this clinical trial?*”—was difficult to answer. Change for each domain-level was more easily and accurately recalled if separated from other concepts (e.g. combining concepts such as change in physical functioning with emotional wellbeing). Based on this feedback, the CaGI-C document was revised and six separate domain-level items were added: symptoms, physical ability, ability to perform ADL, social life, emotions and mental wellbeing, and overall health since the start of the trial. A response option for each level of change, based on the predefined seven-point scale, for each domain-level item was also included. During a final interview, one caregiver reflected on the six domain-level items and instruction document and provided positive feedback on the revisions (see Table 6). Only minor changes to the wording were made to improve understanding. Final CGI-C and CaGI-C items are detailed in Additional file 2 and Additional file 3.

## Discussion

Insights were gathered from clinicians, caregivers and individuals with Duchenne to understand which symptoms and functional impacts of the disease were important and clinically meaningful. This study further confirmed the significance in obtaining the patient’s perspective in rare diseases where heterogeneity exists and the concepts and level of change meaningful for patients and their families can vary [22]. Consistent with published literature, the most frequently experienced symptom reported in all three populations was muscle weakness [23–28]. Clinicians described fatigue and cardiac difficulties as the next most frequently experienced symptoms, while caregivers and individuals with Duchenne reported fatigue and pain.

The findings related to difficulties with physical functioning were consistent amongst the three populations and with information documented in the literature regarding limitations in motor function [26, 27, 29]. All three populations noted that small changes in functional ability were meaningful, particularly when the changes led to a loss or gain of independence. Clinicians frequently described how changes in speed, duration or endurance would be important to an individual with Duchenne and explained that any changes in the quality of movement (e.g. exhibiting less toe-walking) would also be significant. Caregivers also reported that changes in speed, endurance and the quality of movement were important to study participants. Any difference in the levels of effort and confidence of the individual with Duchenne was meaningful to all populations, and any improvement in the abilitiy to keep up with peers was particularly important to caregivers and individuals.

While existing outcome measures (e.g. North Star Ambulatory Assessment) and timed functional tests (e.g. the 6-Minute Walk Test and the Four-Stair Climb Velocity Test) capture relevant concepts to clinicians, patients and their families, subtle changes in physical functioning that families also find meaningful may be hard to detect [30, 31]. Therefore, additional outcomes such as a global impression item, patient and observer-reported outcomes, or more creative methods such as patient videos of functioning or wearable devices, [32] in conjunction with motor function tests may be useful to evaluate change in an interventional setting [22].

A unifying theme relating to meaningful change amongst the three populations was the reduction in the level of assistance necessary to complete an ADL independently. Given that any change in the ability to complete an ADL was significant to families, patient-reported outcomes assessing these concepts should be considered for inclusion as endpoints in clinical trials.

Overall, both the CGI-C and CaGI-C items and instructions were well understood by participants. The descriptions and examples developed from the CE interviews were reported to be relevant and appropriate for illustrating different levels of meaningful change in Duchenne, supporting the content validity of the documents. Clinicians reported that an instruction document would add clarity and consistency in ratings between clinicians in a clinical trial setting. Feedback from clinicians regarding the length of the document led to the separation of the CGI-C into two documents: one containing the item and a short set of instructions and a second, more detailed training document. Feedback from caregivers and individuals with Duchenne placed substantial emphasis on the complexity of the components of HRQoL, cognitive and behavioral functioning, and impact on physical functioning. To address the feedback, the single global question on the overall health of the individual with Duchenne was modified to include six domain-level questions.

While this study provided insight into the experience of Duchenne and what constitutes a meaningful change from the perspective of clinicians, caregivers and individuals with Duchenne, caution should be taken in drawing conclusions from this research due to the limited sample size of each subgroup. While the CE findings provided valuable qualitative perspectives regarding which HRQoL domains were meaningful and important to measure, qualitative insights should ideally be triangulated with statistical distribution and anchor-based quantitative analyses when interpreting clinical outcome assessments [14]. This will ensure that the selection of responder definitions to aid meaningful interpretation of change are sound from a statistical perspective.

## Conclusions

The findings of this study offer valuable insights into changes important for ambulant individuals with Duchenne, and support the initial content validity of the global impression of change items that were drafted, revised and finalized. These assessments are intended to assist clinicians and caregivers to rate clinically meaningful change over the course of a clinical trial. As part of a holistic measurement strategy, such clinical outcome assessments can be incorporated into the clinical trial setting to assess global changes in symptoms and functional impacts associated with Duchenne.

## Supplementary Information

**Additional file 1. ** Table S1 (Most frequently reported meaningful improvements and worsening of selected physical functioning activities in the concept elicitation interviews from the perspective of clinicians, caregivers, and individuals with Duchenne).

**Additional file 2.** Figure S1 (Final clinician rating of change (CGI-C) for Duchenne Muscular Dystrophy: Item and instructions for raters completing the assessment)

**Additional file 3.** Figure S2 (Final caregiver rating of change (CaGI-C) for Duchenne muscular dystrophy: Item and instructions for raters completing the assessment).

## Data Availability

Data generated from this study are not publicly available; additional data may be provided by the authors upon reasonable request.

## Abbreviations

ADL: Activities of daily living
CaGI-C: Caregiver Global Impression of Change
CD: Cognitive debriefing
CE: Concept elicitation
CGI-C: Clinical Global Impression of Change
COA: Clinical outcome assessments
DMD: Duchenne muscular dystrophy
FDA: US Food and Drug Administration
HRQoL: Health-related quality of life
