# Supplementary material for: Development of a Clinical Global Impression of Change (CGI-C) and a Caregiver Global Impression of Change (CaGI-C) measure for ambulant individuals with Duchenne muscular dystrophy
Source: Health Qual Life Outcomes. 2021 Jul 26;19:184. doi: 10.1186/s12955-021-01813-w (PMC8314490; doi:10.1186/s12955-021-01813-w)
Supplement: Supplementary file 1 — Additional file 1. Table S1 (Most frequently reported meaningful improvements and worsening of selected physical functioning activities in the concept elicitation interviews from the perspective of clinicians, caregivers, and individuals with Duchenne). [file 12955_2021_1813_MOESM1_ESM.docx]

**Supplementary Table 1.** Most frequently reported meaningful improvements and worsening of selected physical functioning activities in the concept elicitation interviews from the perspective of clinicians, caregivers, and individuals with Duchenne.

| Physical function | Most frequently reported meaningful improvements  (n, number of participants) | Most frequently reported meaningful worsening  (n, number of participants) |
| --- | --- | --- |
| Walking | **Clinicians**   - Walking longer distances without becoming fatigued (n=5) - Having a normal heel strike (n=3)   *“Of* ***course, the feet are not being carried or dragged****. They’re—they go to the floor or touch the floor easily.”* | **Clinicians**   - Requiring more assistance from supportive devices such as walking frames and devices (n=4) - Walking shorter distances, at slower speeds and walking on toes (n=2)   *“****Slow, slower walking****,* ***having to hold on to something****. I think that would be the main thing.* ***They’d be much more on their toes. They’d be completely unable to get their heels down****. And they would, um,* ***want to hold on to something to maintain their stability and balance****.”* |
|  | **Caregivers & Individuals**   - Walking longer distances without becoming fatigued (n=5; 4 caregivers, 1 individual) - To fall less frequently when walking (n=3, 2 caregivers and 1 individual)   *“Um, the, the walking would be* ***easier****, so I wouldn’t get tired and I* ***won’t fall****.”* | **Caregivers**   - Feeling different to peers (n=2 caregivers) - Walking more slowly, requiring more effort, relying on assistive devices, postural changes, increased toe walking, wider stance and losing   the ability to walk (all raised by n=1 caregiver)  *“Probably needing to use the* ***scooter*** *at school on a regular basis.”* |
| Climbing stairs | **Clinicians**   - Climbing the stairs faster and in less time (n=3)   *“****If they take less time than they previously did****, then it is a clinically significant improvement.”*   - Ability to climb the stairs without falling or losing balance and requiring less effort to climb stairs (n=1) | **Clinicians**   - Needing more assistance from others to climb stairs (n=2) - Ability to climb only one step or none and requiring more time than previously to climb stairs (n=1)   *“If they’re* ***needing assistance*** *or if they’re* ***falling down****, then it is a clinically significant regression.* |
|  | **Caregivers & Individuals**   - Climbing stairs more quickly (n=2 caregivers, 1 individual) - Ascending with less assistance, (e.g. less use of the banister) (n=3 caregivers) - Requiring less effort to climb the stairs (n=3; 2 caregivers and one individual)   *“I don’t know. Just to do better. You know,* ***just have more energy, have more strength****.”* | **Caregivers**   - Losing the ability to ascend/descend (n=4; 4 caregivers)   *“That he* ***couldn’t do it at all*** ***even with a lot of assistance****.”*   - Needing to crawl up the stairs (n=3; 3 caregivers) |
| Stand from sitting on a chair | **Clinicians**   - Requiring little to no arm support to sit up (n=2)   *“If they—obviously* ***if they can get up without using their arms****. Or if they can get up maybe, you know, using the arms just a bit to, to balance themselves, not really to push off.*   - Standing up faster than before and standing without external assistance (n=1) | **Clinicians**   - Losing the ability to get up from a seated position and needing assistance to stand (n=3 for both concepts)   *“N****eeding somebody to help them rise from a chair*** *having somebody kind of pull them up to a standing position.”* |
|  | **Caregivers & Individuals**   - Ability to stand with less effort, having more confidence that their legs would hold their weight, being able to more consistently stand up from chair, not falling back into chair and standing up from a higher chair (n=1 for all concepts)   *“Yeah, just having the* ***confidence*** *to put his feet down, I think that his body's going to hold him.”* | **Caregivers & Individuals**   - Not being able to stand up (n=3; 3 caregivers) - Requiring more effort to stand up (n=3; 2 caregivers, 1 individual)   *“****Not being able to*** *or, you know,* ***struggling a lo****t would be bad.”* |
| Standing up from floor | **Clinicians: stand up from lying or sitting on the floor**   - Standing up with less involvement of all four limbs (Gowers’ movement) (n=4) - Ability to stand up independently, without assistance from others and standing up faster (n=3)   *“From an individual’s point of view* ***being able to do that independently*** *and again in a way that* ***you’re not so daunted by doing*** *it that you dread it… again it’s another element of independence.”* | **Clinicians: stand up from lying or sitting on the floor**   - Needing partial or full assistance to stand up from the floor and taking longer to stand up or to carry out this action very slowly (n=3 for each concept)   *“Worsening would be they would keep their hands on the floor for a longer period of time…”It would just be a much slower process.”* |
|  | **Caregivers & Individuals: stand up from lying on the floor**   - Using less effort to stand up (n=4; 2 caregivers and 2 individuals) - Ability to sit up without rolling onto stomach (n=3; 3 caregivers)   *“I guess would be I think probably to help him a little bit would be to actually be able to* ***sit up without having to turn over.****”* | **Caregivers: stand up from lying on the floor**   - Losing ability to pull up to sit, requiring more effort to sit, losing the ability to stand up from lying, trunk weakness (n=1 caregiver for all concepts)   *“****Not being able to do*** *that [stand up from lying] would be way worse.”* |
