# Supplementary material for: Development of a Clinical Global Impression of Change (CGI-C) and a Caregiver Global Impression of Change (CaGI-C) measure for ambulant individuals with Duchenne muscular dystrophy
Source: Health Qual Life Outcomes. 2021 Jul 26;19:184. doi: 10.1186/s12955-021-01813-w (PMC8314490; doi:10.1186/s12955-021-01813-w)
Supplement: Supplementary file 2 — Additional file 2. Figure S1 (Final clinician rating of change (CGI-C) for Duchenne Muscular Dystrophy: Item and instructions for raters completing the assessment) [file 12955_2021_1813_MOESM2_ESM.docx]

Supplementary material 2. Final clinician rating of change (CGI-C) for Duchenne Muscular Dystrophy: Item and instructions for raters completing the assessment

**Duchenne Muscular Dystrophy (Duchenne) Clinical Global Impression of Change (CGI-C): Item and instructions for raters completing the assessment**

The Duchenne CGI-C is a single-item assessment, intended to measure change in the clinical status (symptoms and functional ability) of individuals with Duchenne, from baseline until a later pre-defined time point in the clinical trial.

**Information to consider when using the CGI-C to assess change**

As the CGI-C is a global assessment, you should consider the individual’s clinical status (symptoms and functional ability) when rating the degree of change. This includes but is not limited to:

- Symptoms such as:
  - Muscle strength
  - Pain
  - Fatigue
- Motor functioning/physical functioning
- Ability to perform daily activities
- Adverse events

When making your rating, information relating to clinical status (symptoms and functional ability) should be collected based on all of the information available (e.g., outcome measures, interactions with caregivers and individuals). Even small changes in symptoms or motor functioning may lead to a meaningful improvement or worsening in the patient’s clinical status.

**Taking into account all aspects of the individual’s Duchenne symptoms and functional ability, how would you rate the change in clinical status for this individual since the start of the study? Please select one response only.**

| Response option | Change category | Description |  |
| --- | --- | --- | --- |
| 1 | Very much improved | Individual has shown a very large improvement in their clinical status (symptoms and functional ability), which has had a meaningful impact on their daily life or wellbeing. | ☐ |
| 2 | Much improved | Individual has shown several small improvements or a large improvement in their clinical status (symptoms and functional ability), which has had a meaningful impact on their daily life or wellbeing. | ☐ |
| 3 | Minimally improved | Individual has shown small improvements in their clinical status (symptoms and functional ability), which has had a meaningful impact on their daily life or wellbeing. | ☐ |
| 4 | No change | Individual has not experienced any noticeable changes in clinical status (symptoms and functional ability) which has meant daily life and wellbeing has been maintained. | ☐ |
| 5 | Minimally worse | Individual has shown small declines in their clinical status (symptoms and functional ability), which has had a meaningful impact on their daily life or wellbeing. | ☐ |
| 6 | Much worse | Individual has shown several small declines or one large decline in their clinical status (symptoms and functional ability), which has had a meaningful impact on their daily life or wellbeing. | ☐ |
| 7 | Very much worse | Individual has shown a very large decline in their clinical status (symptoms and functional ability), which has had a meaningful impact on their daily life or wellbeing. | ☐ |

*The CGI-C for Duchenne Muscular Dystrophy can be accessed and licensed via* [*Mapi Research Trust*](https://eprovide.mapi-trust.org/instruments/duchenne-muscular-dystrophy-clinical-global-impression-of-change)*.*
