# Supplementary material for: Development of a Clinical Global Impression of Change (CGI-C) and a Caregiver Global Impression of Change (CaGI-C) measure for ambulant individuals with Duchenne muscular dystrophy
Source: Health Qual Life Outcomes. 2021 Jul 26;19:184. doi: 10.1186/s12955-021-01813-w (PMC8314490; doi:10.1186/s12955-021-01813-w)
Supplement: Supplementary file 3 — Additional file 3. Figure S2 (Final caregiver rating of change (CaGI-C) for Duchenne muscular dystrophy: Item and instructions for raters completing the assessment). [file 12955_2021_1813_MOESM3_ESM.docx]

**Supplementary Figure 2.** Final caregiver rating of change (CaGI-C) for Duchenne Muscular Dystrophy: Item and instructions for raters completing the assessment

**Caregiver rating of change for Duchenne Muscular Dystrophy (Duchenne)**

**Purpose of these instructions**
This document provides instructions to help you to rate the change you have observed since the start of the clinical trial in the child with Duchenne who you care for. These instructions were developed using information from interviews with children aged 8-11 years old with Duchenne and caregivers of children with Duchenne aged 6-11 years old.

**How to rate any changes that were experienced by the child during the clinical trial**On the next page, there are six separate questions for you to answer about any changes in the child’s: (1) symptoms, (2) physical ability, (3) ability to perform daily activities, (4) social life, (5) emotions and mental wellbeing and (6) overall health since the start of the clinical trial.

The table below gives descriptions for each level of change to help you think about each response option and any changes you have seen in the child that you care for. Thinking about the descriptions below please rate the level of change for questions 1-6 on the next page.

| Change category | Description |
| --- | --- |
| Very much worse (7) | The child has shown a very large amount of worsening in either their symptoms, physical ability, ability to perform daily activities, social life, emotions and mental wellbeing or overall health, which has had a large and important impact on their life. |
| Much  worse (6) | The child has shown a medium to large amount of worsening in either their symptoms, physical ability, ability to perform daily activities, social life, emotions and mental wellbeing or overall health, which has had a moderate and important impact on their life. |
| Minimally worse (5) | The child has shown a small amount of worsening in either their symptoms, physical ability, ability to perform daily activities, social life, emotions and mental wellbeing or overall health, which has had a small and important impact on their life. |
| No change (4) | The child has not experienced any noticeable change in either their symptoms, physical ability, ability to perform daily activities, social life, emotions and mental wellbeing or overall health. |
| Minimally Improved (3) | The child has a shown small amount of improvement in either their symptoms, physical ability, ability to perform daily activities, social life, emotions and mental wellbeing or overall health, which has had a small and important impact on their life. |
| Much improved (2) | The child has shown a medium to large amount of improvement in either their symptoms, physical ability, ability to perform daily activities, social life, emotions and mental wellbeing or overall health, which has had a moderate and important impact on their life. |
| Very much improved (1) | The child has shown a very large amount of improvement in either their symptoms, physical ability, ability to perform daily activities, social life, emotions and mental wellbeing or overall health, which has had a large and important impact on their life. |

For the following questions, please indicate how much change the child that you care for has experienced between starting this trial and today. Please choose one response per question.

| 1. Comparing the start of the clinical trial to now, how would you rate any changes in the child’s symptoms (e.g. strength, pain, tiredness)? Choose only one response. | | | | | | |
| --- | --- | --- | --- | --- | --- | --- |
| Very much worse  □ | Much worse  □ | Minimally worse  □ | No Change  □ | Minimally improved  □ | Much improved  □ | Very much improved  □ |

| 1. Comparing the start of the clinical trial to now, how would you rate any changes in the child’s physical ability (e.g. walking, climbing stairs, standing up from a chair)? Choose only one response. | | | | | | |
| --- | --- | --- | --- | --- | --- | --- |
| Very much worse  □ | Much worse  □ | Minimally worse  □ | No Change  □ | Minimally improved  □ | Much improved  □ | Very much improved  □ |

| 1. Comparing the start of the clinical trial to now, how would you rate any changes in the child’s ability to perform daily activities (e.g. dressing, bathing/showering, eating and drinking)? Choose only one response. | | | | | | |
| --- | --- | --- | --- | --- | --- | --- |
| Very much worse  □ | Much worse  □ | Minimally worse  □ | No Change  □ | Minimally improved  □ | Much improved  □ | Very much improved  □ |

| 1. Comparing the start of the clinical trial to now, how would you rate any changes in the child’s social life (e.g. interactions with friends and family and taking part in social activities)? Choose only one response. | | | | | | |
| --- | --- | --- | --- | --- | --- | --- |
| Very much worse  □ | Much worse  □ | Minimally worse  □ | No Change  □ | Minimally improved  □ | Much improved  □ | Very much improved  □ |

| 1. Comparing the start of the clinical trial to now, how would you rate any changes in the child’s emotions and mental wellbeing (e.g. mood, confidence, anxiety)? Choose only one response. | | | | | | |
| --- | --- | --- | --- | --- | --- | --- |
| Very much worse  □ | Much worse  □ | Minimally worse  □ | No Change  □ | Minimally improved  □ | Much improved  □ | Very much improved  □ |

| 6a. Comparing the start of the clinical trial to now, thinking about the child’s symptoms, physical ability, ability to perform daily activities, social life and emotions and mental wellbeing, how would you rate any changes in the child’s overall health? Choose only one response. | | | | | | |
| --- | --- | --- | --- | --- | --- | --- |
| Very much worse  □ | Much worse  □ | Minimally worse  □ | No Change  □ | Minimally improved  □ | Much improved  □ | Very much improved  □ |

| 6b. Is the level of change that you selected for question 6 important to you? | |
| --- | --- |
| Yes □ | No □ |

*The CaGI-C for Duchenne Muscular Dystrophy can be accessed and licensed via* [*Mapi Research Trust.*](https://eprovide.mapi-trust.org/catalog)
